# Intraoperative patellar maltracking and postoperative radiographic patellar malalignment were more frequent in cases of complete medial collateral ligament release in cruciate-retaining total knee arthroplasty

**DOI:** 10.1186/s43019-021-00091-6

**Published:** 2021-03-20

**Authors:** Jung Ho Noh, Nam Yeop Kim, Ki Ill Song

**Affiliations:** 1 Department of Orthopaedic Surgery, Kangwon National University School of Medicine, 1 Gangwondaehak-gil, Chuncheon-si, Gangwon-do 24341 South Korea; 2 Department of Orthopaedic Surgery, Kangwon National University Hospital, Chuncheon-si, South Korea

**Keywords:** Patella, Total knee arthroplasty, Cruciate retaining, Tracking

## Abstract

**Background:**

Patellar maltracking after total knee arthroplasty (TKA) can lead to significant patellofemoral complications such as anterior knee pain, increased component wear, and a higher risk of component loosening, patellar fracture, and instability. This study was to investigate the preoperative and operative variables that significantly affect patellar tracking after cruciate-retaining TKA.

**Methods:**

We studied 142 knee joints in patients who had undergone TKA: the knees were dichotomized based on postoperative patellar tracking, which was evaluated on patellar skyline, axial-projection radiographs: group 1, normal patellar tracking (lateral tilt ≤ 10° and displacement ≤ 3 mm) and group 2, patellar maltracking (lateral tilt > 10° or displacement > 3 mm). The patients’ demographic data and clinical and radiographic measurements obtained before and after surgery were compared between the two groups.

**Results:**

Preoperative lateral patellar displacement was greater (4.1 ± 2.6 mm vs. 6.0 ± 3.5 mm), as was the frequency of medial collateral ligament (MCL) release (3/67 vs. 24/75) in group 2 than in group 1 (*p* < 0.001 and *p* < 0.001, respectively). The distal femur was cut in a greater degree of valgus in group 1 than in group 2. (6.3 ± 0.8° vs. 6.0 ± 0.8°) (*p* = 0.034).

**Conclusions:**

Complete release of the MCL during surgery was associated with patellar maltracking (logistic regression: *p* = 0.005, odds ratio = 20.592). Surgeons should attend to patellar tracking during surgery in medially tight knees.

**Level of evidence:**

Retrospective comparative study, level III.

## Introduction

Patellar maltracking after total knee arthroplasty (TKA) can lead to anterior knee pain, increased component wear, and a higher risk of component loosening, patellar fracture, instability, and poor clinical outcomes [1–3]. Surgeons have attempted to reduce the incidence of symptomatic patellar tilt and subluxation by modifying their surgical technique, including use of the quadriceps-sparing approach, prevention of internal rotation positioning of the femoral and tibial components, medialization of the patellar component, and release of the lateral patellar retinaculum. However, optimizing patellar tracking is sometimes a challenge. Identifying and correcting patellar maltracking intraoperatively may not only decrease the rate of anterior knee pain and other patellar complications but also avoid the need for revision surgery.

Soft tissue release is often necessary to obtain optimal tibiofemoral ligament balance in TKA. However, extensive release of soft tissue such as the medial collateral ligament (MCL) can implicate physiological femorotibial rotational movement [4], which may also affect patellofemoral articulation. The purpose of this study was to investigate various preoperative and operative variables that could significantly affect patellar tracking postoperatively. The null hypothesis was that no factor or combination of factors significantly influenced patellar tracking after TKA.

## Materials and methods

### Subjects

**Fig. 1:**
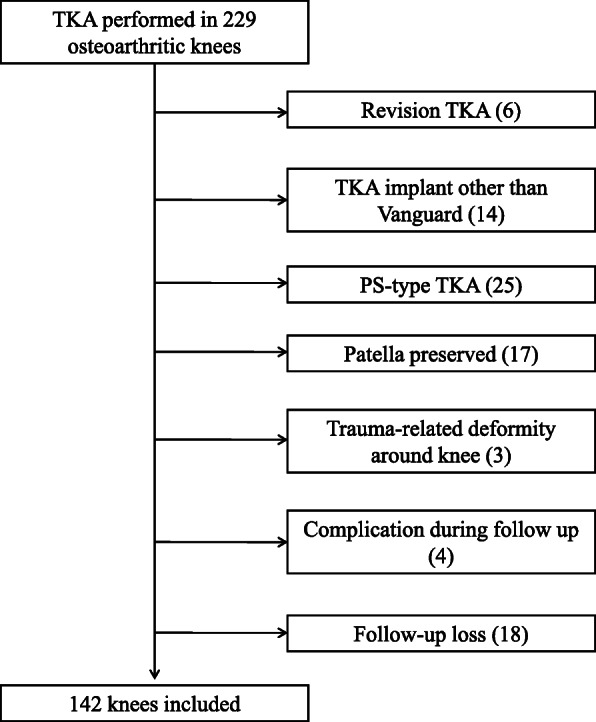
Flow diagram of patient selection. TKA, total knee arthroplasty; PS, posterior cruciate ligament (PCL)-substituting

This retrospective study was performed with the approval of the institutional review board of the hospital. An experienced surgeon performed TKA on 229 osteoarthritic knees (185 patients) between 2012 and 2014. Primary TKA was performed using the Vanguard Complete Knee system (Biomet, Warsaw, IN, USA) in 209 of the 229 knees (172 patients). When the knee was severely deformed or the posterior cruciate ligament (PCL) was significantly degenerated, attenuated, or absent, the PCL-substituting (PS) type of TKA was performed. Otherwise, the cruciate-retaining (CR) type of TKA was performed. The inclusion criterion was primary CR-TKA using the Vanguard Complete Knee for the treatment of osteoarthritis. The exclusion criteria were TKA with an implant other than the CR-type Vanguard Complete Knee system, follow up for less than 3 years, TKA without patellar resurfacing, preoperative presence of significant trauma-related intraarticular or extraarticular bony deformity around the knee, or revision TKA. Four patients who had complications during follow up were also excluded. One of these patients had a periprosthetic fracture of the distal femur, two had avascular necrosis of the patella, and one underwent secondary arthroscopic synovectomy due to impingement of the synovium after TKA. Ultimately, a total of 142 knees were studied in 111 patients (21 men (21 knees) and 90 women (121 knees), mean age 70.5 years (range 53–87 years)), who received CR-TKA with patellar resurfacing (Fig. 1).

### Surgical technique

The operation was performed by an experienced surgeon (JHN) using a uniform approach and technique and the CR-type Vanguard Complete Knee system. Surgery was usually performed under spinal anesthesia, and a tourniquet was applied in all cases. The surgeon performed an anterior midline skin incision and medial parapatellar arthrotomy. Intramedullary instrumentation was used to properly align the distal femoral cut, which was set perpendicular to the mechanical axis of the femur. The valgus angle for the distal femoral cut was determined from a preoperative, long-leg, standing anteroposterior (AP) projection radiograph with the patellae facing forward, and verified using the extramedullary instrumentation guide during surgery. The measured resection technique was used to determine the extent of the distal femoral resection. An anterior referencing guide was used to determine the size of the femoral component. If the distal femur was between sizes, a smaller femoral component size was chosen. Rotation of the AP femoral cut was set to be perpendicular to Whiteside’s line. The rotation angle of the femoral cut from the posterior condylar line was recorded. The femoral component was lateralized as far as there was no impingement between the PCL and the femoral component, and lateral overhang was avoided when establishing the mediolateral position.

The proximal tibial cut was set perpendicular to the mechanical axis of the tibia. Tibial component rotation was determined by referencing the tibial AP axis, which was defined as a line extending from the center of the tibial footprint of the PCL to the medial one third of the tibial tuberosity with the AP axis of the tibial component parallel to the tibial AP axis.

When the knee was tight medially in extension, the deep MCL was released using a curved osteotome and the posteromedial capsule was released using electrocautery to the posterior border of the superficial MCL. If the knee was still tight, the posterior half of the superficial MCL was subperiosteally released. When the knee was tight medially at 90° of flexion, the deep MCL was completely released and the distal portion of the superficial MCL was subperiosteally released below the joint line, step by step in 1-cm increments, using a curved osteotome, until the ligament balance was appropriate. Appropriate ligament balance was defined as < 2 mm difference between the medial and lateral gaps, under manual stress with the trial components in place. Complete release of the MCL was sometimes required in severe contraction of the medial soft tissue. Complete release of the MCL was defined by sudden widening of the medial gap with complete loss of MCL tension on palpation. The PCL was released at the tibial attachment when the knee was tight in flexion or when the anterior tibial tray of the implant lifted off as the knee was flexed.

The standard patellar resurfacing technique was used to restore preoperative patellar thickness and maintain symmetrical bone resection. The dome-shaped patellar component was placed in the center of the patellar articular surface. Postoperative patellar thickness was set to be the same as or within 1 mm less than the preoperative patellar thickness. However, the patella was resected with the remainder not < 12 mm thick in all cases in case of a thin patella. Patellar thickness was measured by a caliper before and after resurfacing and recorded during surgery.

After implanting all components with cement, tracking of the patellofemoral articulation was assessed using the “no thumb technique” throughout the entire range of motion. Any subluxation, dislocation, or visible elevation of the medial edge of the patellar component was considered a positive patellar tracking test. In these cases, patellar tracking was assessed again with the tourniquet deflated. If the patellar maltracking persisted, the lateral retinaculum was released to optimize patellar tracking and minimize patellar tilt. The lateral retinaculum was uniformly released from the inside out, approximately 1 cm lateral to the lateral margin of the patella. Usually, only a short, distinct, and thickened 2-cm band was released. The surgeon attempted to preserve the lateral geniculate artery during release, and this was cauterized if cut. The arthrotomy was repaired with simple interrupted sutures using Vicryl 2.0.

Quadriceps set exercises were started immediately after surgery. Continuous knee motion exercises and walking with crutches were started on postoperative day 2.

### Assessment of outcomes

**Fig. 2:**
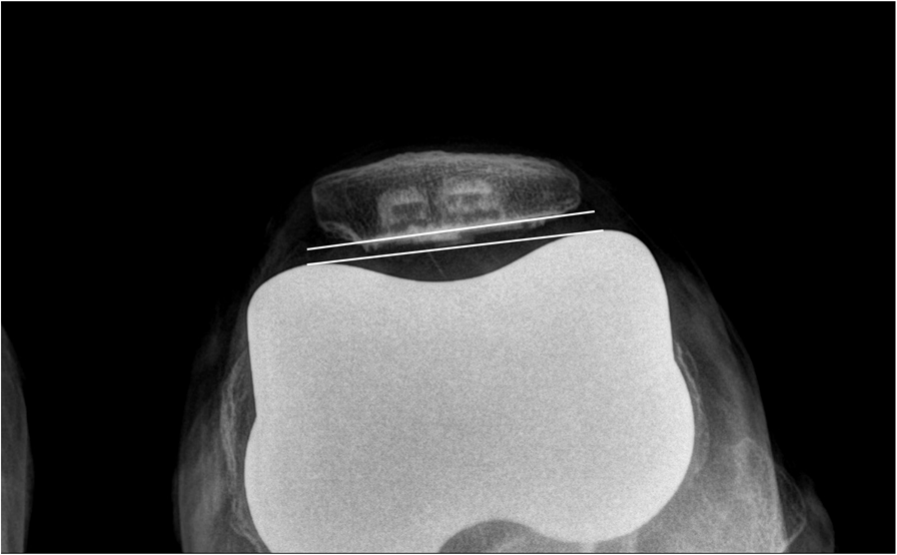
Postoperative patellar tilt was defined as the angle between the intercondylar line and a line drawn through the prosthesis-bone interface. This skyline axial-projection radiograph shows a patellar tilt of 1.3°, which corresponds to normal patellar tracking

**Fig. 3:**
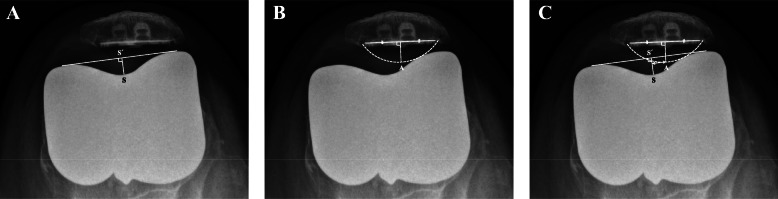
**a** A perpendicular line (SS´) from the deepest point of the sulcus (S) is drawn to a line connecting the most anterior aspects of the medial and lateral femoral trochlear facets. **b** Apex of the patellar dome (A) is marked, which is on the perpendicular bisector of the base. **c** Patellar displacement is defined as the distance between point A and line SS´

**Fig. 4:**
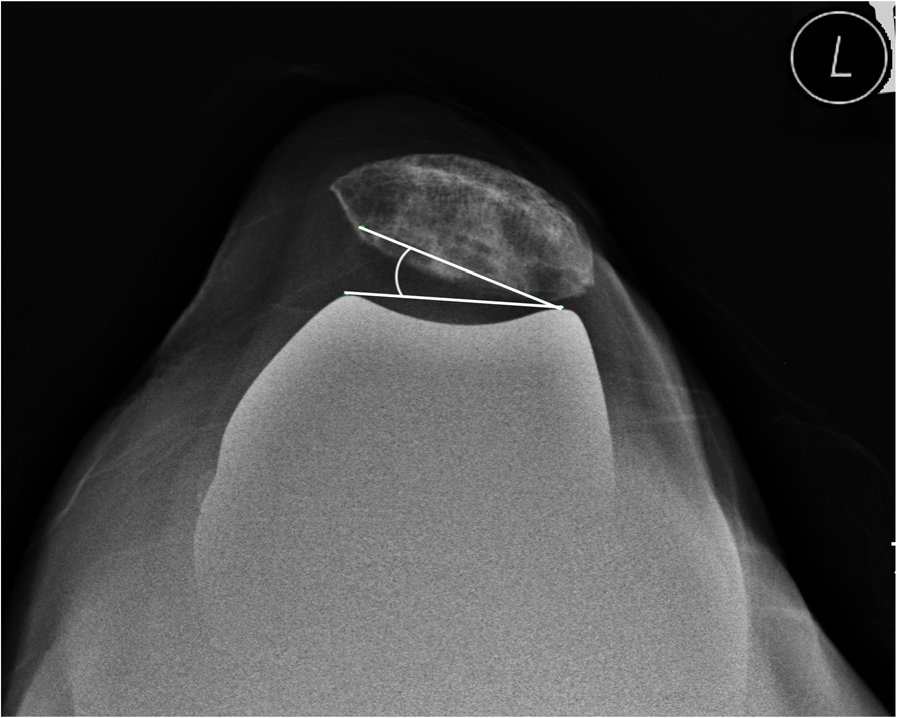
A skyline axial-projection radiograph shows lateral patellar tilt of 16.8° corresponding to patellar maltracking

**Fig. 5:**
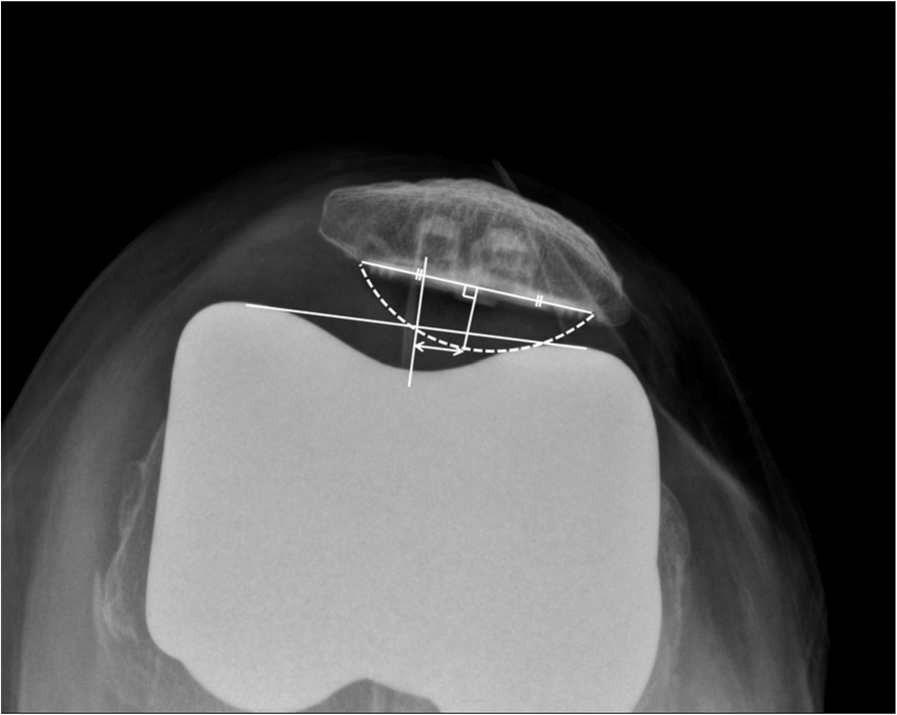
A skyline axial-projection radiograph shows lateral patellar displacement of 6.5 mm corresponding to patellar maltracking

The patients’ preoperative data, including age, sex, height, weight, and body mass index (BMI, kg/m^2^) were assessed. Range of motion (ROM) of the knee and Knee Society scores were evaluated using a goniometer preoperatively and at the last follow up. Patellar thickness before and after resurfacing was assessed intraoperatively. All subjects were radiographically evaluated on standing AP-projection, lateral-projection, skyline axial-projection, and long-leg standing, AP-projection radiograph preoperatively, postoperatively, and at the last follow up. Postoperative radiographs were acquired after stitches were removed on postoperative day 14. Skyline axial radiographs of the patella were acquired with the knee in approximately 30° flexion [5]. Lower extremity alignment was measured from long-leg standing, AP-projection radiographs. Preoperative patellar tilt and displacement were measured using skyline axial-projection radiographs, as described by Kim et al. [6]. Patellar tilt was defined as the angle between the equatorial line of the patella and the anterior intercondylar line. Patellar displacement was defined as the distance from the intercondylar sulcus to the apex of the patella, which is the deepest point of the patella in relation to the equatorial line of the patella. Lateral patellar tilt or displacement was presumed to be positive. Postoperative patellar tilt and patellar displacement were measured as described by Nagai et al. [5] (Figs. 2, 3, 4, 5). The Insall–Salvati ratio [7] and Blackburne-Peel ratio [8] were measured on lateral-projection radiographs obtained with knee flexion of 30°. Patellar tilt > 10° or patellar displacement > 3 mm was considered positive for patellar maltracking [9]. Patellar maltracking was identified when the lateral retinaculum was released during surgery (31 cases), regardless of the postoperative radiographic assessment. The knees were dichotomized into group 1, normal patellar tracking (67 cases) or group 2, patellar maltracking (75 cases). Patient factors (sex, height, weight, BMI, degree of flexion contracture of the knee, preoperative knee ROM, preoperative lower limb alignment, preoperative patellar tilt, preoperative patellar displacement, preoperative Insall–Salvati ratio, preoperative Blackburne-Peel ratio, and patellar thickness before resurfacing) and surgical factors (whether or not there was complete release of the superficial MCL or of the PCL, the valgus angle of the distal femoral cut, and the rotation angle of the distal femoral cut) were compared between the groups to determine which factors affected postoperative patellar tracking.

### Statistical analyses

Data were analyzed using SPSS ver. 17.0 software (SPSS Inc., Chicago, IL, USA). Mean and standard deviation were used to describe the data. Two orthopedic surgeons performed the radiographic measurements. Interobserver reliability for radiographic measurements was assessed by the intraclass correlation coefficient (ICC) with 95% confidence interval. The ICCs were > 0.85 for all parameters, and the values measured by the senior surgeon were used for statistical analyses. Data were compared between the groups using Student’s *t* test, repeated measured analysis of variance (ANOVA), and the chi-square test. Logistic regression was conducted to determine the risk factors for patellar maltracking. Postoperative patellar tracking was compared to that at the last follow up using the dependent *t* test. *P* values <0.05 were considered significant. The power of this study is 0.8 in post hoc analysis.

## Results

Table 1 shows the patient and surgical factors in the two groups. Mean knee ROM was 132.2 ± 13.4° preoperatively and 130.2 ± 13.1° at the last follow up (*p* = 0.104). Mean Knee Society knee score was 47.1 ± 5.0 preoperatively and 91.4 ± 5.1 at the last follow up (*p* < 0.001). Mean Knee Society function score was 28.4 ± 11.9 preoperatively and 78.9 ± 7.9 at the last follow up (*p* < 0.001). Mean patellar tilt was 3.7 ± 3.4°, and mean patellar displacement was 5.1 ± 3.3 mm, preoperatively. In group 2, patellar maltracking remained apparent on postoperative radiography in 5 of 31 cases of lateral retinacular release during surgery. Knee ROM and Knee Society scores are compared between the groups in Table 2. Table 3 shows the results of univariate regression analysis of the risk factors for postoperative patellar maltracking. Postoperative patellar maltracking was a risk factor for medial release during surgery (*p* = 0.005), and the odds ratio was 20.592. Mean patellar displacement after surgery was significantly different to that at the last follow up (*p* = 0.011), but mean patellar tilt was not (*p* = 0.100) (Table 4).

**Table 1:** Comparisons of patient and surgical factors between groups

	Group 1^a^ (*n* = 67)	Group 2^b^ (*n* = 75)	*P* value
Age (years)	72.1 ± 7.4	69.1 ± 7.1	0.015^c^
Sex (male:female)	12:55	9:66	0.352^d^
Height (cm)	152.1 ± 8.6	151.1 ± 6.2	0.421^c^
Weight (kg)	59.9 ± 10.9	63.0 ± 8.5	0.061^c^
BMI (kg/m^2^)	25.4 ± 4.8	27.6 ± 3.3	0.002^c^
Preoperative flexion contracture (degrees)	4.3 ± 8.8	5.2 ± 8.6	0.551^c^
Preoperative further flexion (degrees)	137.0 ± 7.6	135.9 ± 9.4	0.427^c^
Preoperative mechanical axis (degrees)	Varus 8.4 ± 6.6	Varus 8.2 ± 5.9	0.855^c^
Postoperative mechanical axis (degrees)	0.5 ± 2.1	0.4 ± 2.0	0.752^c^
Preoperative patellar tilt (degrees)	3.7 ± 3.3	3.8 ± 3.6	0.876^c^
Postoperative patellar tilt (degrees)	2.3 ± 3.4	5.7 ± 4.5	0.017^c^
Preoperative patellar displacement (mm)	4.1 ± 2.6	6.0 ± 3.5	<0.001^c^
Postoperative patellar displacement (mm)	1.1 ± 1.5	4.0 ± 2.2	<0.001^c^
Preoperative Insall-Salvati ratio	0.9 ± 0.1	0.9 ± 0.1	0.520^c^
Preoperative Blackburne-Peel ratio	1.3 ± 0.1	1.5 ± 0.2	0.108^c^
Preoperative patellar thickness (mm)	21.5 ± 2.0	21.5 ± 1.5	0.832^c^
Postoperative patellar thickness (mm)	21.4 ± 1.5	21.3 ± 1.2	0.711^c^
MCL release (yes:no)	3:64	24:51	<0.001^d^
PCL release (yes:no)	10:57	15:60	0.511^d^
Femoral valgus cut angle (degrees)	6.3 ± 0.8	6.0 ± 0.8	0.034^c^
Distal femoral cut rotation (degrees)	4.6 ± 1.4	4.5 ± 1.6	0.686^c^

^a^Group 1: normal patellar tracking intraoperatively and postoperatively

^b^Group 2: patellar maltracking intraoperatively or postoperatively

^c^Student *t* test

^d^Chi-square test

**Table 2:** Mean range of motion (ROM) and Knee Society scores according to group

	Group 1 (n = 67)	Group 2 (n = 75)	*P* value
Preoperative ROM (degrees)	132.7 ± 13.9	130.7 ± 13.9	0.389^a^
ROM (degrees) at the final follow up	129.9 ± 14.0	130.4 ± 12.2	0.582^b^
Preoperative knee score	47.4 ± 4.7	46.8 ± 5.2	0.453^a^
Knee score at the final follow up	91.5 ± 4.6	91.4 ± 5.6	0.674^b^
Preoperative function score	28.4 ± 11.9	28.3 ± 11.9	0.934^a^
Function score at the final follow up	78.0 ± 7.3	79.8 ± 8.4	0.458^b^

^a^Student t test

^b^Repeated measures analysis of variance

**Table 3:** Logistic regression of risk factors of patellar maltracking

	B	*P* value	Exp (B)
Age	− 0.030	0.558	0.970
Sex	2.341	0.116	10.394
Height	0.089	0.743	1.093
Weight	−0.199	0.568	0.820
BMI	0.468	0.557	1.596
Preoperative flexion contracture	−0.004	0.919	0.996
Preoperative further flexion	0.011	0.815	1.011
Preoperative mechanical axis	0.049	0.355	1.050
Preoperative patellar tilt	0.009	0.936	1.009
Preoperative patellar displacement	0.253	0.075	1.288
Preoperative Insall-Salvati ratio	0.385	0.868	1.470
Preoperative Blackburne-Peel ratio	−4.321	0.059	0.013
Preoperative patellar thickness	0.724	0.053	2.062
MCL release	3.025	0.005	20.592
PCL release	−0.134	0.858	0.874
Femoral valgus cut angle	−0.710	0.074	0.492
Distal femoral cut rotation	−0.052	0.799	0.950

*MCL* medial collateral ligament, *PCL* posterior cruciate ligament

**Table 4:** Mean patellar tilt and patellar displacement

	Postoperative	At the last follow up	*P* value
Mean patellar tilt	3.9 ± 4.7°	4.5 ± 4.2°	0.100^a^
Mean patellar displacement	2.0 ± 2.1 mm	2.6 ± 2.2 mm	0.011^a^

^a^Dependent *t* test

Two knees (one patient) were excluded from analysis; this patient had undergone lateral retinacular release, and had fragmented patellae and collapse corresponding to avascular necrosis 8 months after surgery. The patient had no significant pain and refused revision surgery.

## Discussion

The results of this study revealed that the factor affecting postoperative patellar tracking was whether or not the superficial MCL was released. To be specific, patellar tracking tended to be abnormal postoperatively when the superficial MCL was significantly released during surgery. Rajkumar et al. [10] stated that preoperative femorotibial valgus alignment, patellar tilt, or patellar displacement was associated with postoperative patellar maltracking, whereas distal femoral valgus angle, proximal tibial varus angle, and Insall-Salvati ratio were not associated with postoperative patellar tracking. Some authors have reported that patellar thickness is one of the determinants of patellar tracking [11]. However, most knee surgeons would not intentionally increase patellar thickness during patellar resurfacing. We tried to restore patellar thickness during resurfacing; thus, the assessment of the impact of change in the patellar thickness on patellar tracking may not mean much in this study. This study showed that postoperative patellar tracking was not affected by preoperative patellar thickness or patellar height.

In this study, significant release of the superficial MCL was a risk factor for patellar maltracking. One assumption is that when a tight MCL is released, the tibia is supposed to rotate externally when the knee is flexed with all components implanted and the capsule unrepaired, which makes the patella shift laterally [4]. Although most of the released MCL heals spontaneously, patellofemoral kinematics would not return to the preoperative state because the tension of the healed MCL would not be the same as it was before surgery. Another assumption is that more extensive disruption of the extensor mechanism is required for exposure of more advanced osteoarthritic or severe varus knees, thus with potentially more contraction of the MCL, which may account for patellar maltracking. However, regression analysis revealed that a preoperative femorotibial angle measured on a long-leg, standing, AP-projection radiograph was not a factor affecting postoperative patellar tracking. Regarding this assumption, preoperative valgus-stress radiographs may be helpful to assess whether or not there is contraction of the MCL.

Many studies of the patellofemoral relationship have been based on TKA with femoral component rotation relative to the posterior condylar axis set to the consistent angle, 3° or 5°. We set the femoral component rotation on a case by case basis with Whiteside’s line as a reference, and the femoral component rotation did not affect postoperative patellar tracking. We think that it is more pertinent to consider Whiteside’s line as a reference rather than the posterior condylar axis, to assess patellofemoral articulation [12].

We found that patellar displacement changed as time went on in many cases, but this does not seem to be clinically significant. Our results are supported by those of Ozkoc et al. [13]. However, they stated that the quadriceps-sparing approach was superior to the medial parapatellar approach in terms of late patellar tracking.

Patellar maltracking should be corrected during surgery, as proper patellar tracking is necessary for a successful outcome, and various techniques have been introduced to prevent patellar maltracking, such as preventing internal rotation of the femoral or tibial component and lateral positioning of the components, modifying the implant design, and the quadriceps-sparing approach. Lateral retinacular release is occasionally necessary despite these approaches, with frequencies of 8–45% [14–16]. We performed lateral retinacular release in 21.8% of all cases in this study. Patellar maltracking of most cases in group 2 was not severe as patellar tracking was corrected selectively during surgery, which may explain the outcome of no difference in the postoperative Knee Society scores between the two groups. However, this procedure may cause significant complications, such as avascular necrosis of the patella or patellar fracture [17, 18]. Patella-supporting vascular damage is sometimes inevitable during lateral retinacular release. We observed two cases (one patient) of avascular necrosis during the study period.

This study had several strengths. First, we used a single CR-type implant, and there were no cases of inevitable downsizing of the femoral component to avoid mediolateral overhang of the implant, in which the joint level would have to be changed to balance the flexion and extension gaps. Second, surgery was all performed by one surgeon using a consistent technique. The measured resection technique was used for femoral resection, and a modified measured resection technique with specific instruments was used for tibial resection. Third, patients with deformities related to previous trauma were excluded to minimize the effects of extrinsic factors.

There were a few limitations in this study. First, the number of cases was relatively small. However, we reduced demographic variability by including only osteoarthritic knees. Second, the follow-up period was short. This study focused on assessing patellar tracking, which is a radiographic finding, not a clinical finding. Thus, 3 years may have been adequate to assess these radiographic findings. Third, bone and implant geometry were not investigated. Anterior condylar offset or trochlear depth, which vary considerably and change during TKA even with a single prosthesis design, may affect patellofemoral kinematics [19, 20]. Fourth, rotation of the femoral and tibial components was not assessed. Using a navigation system during surgery or computed tomography postoperatively may be helpful.

## Conclusions

Complete release of the superficial MCL during surgery was associated with patellar maltracking after TKA. Surgeons should attend to patellar tracking during surgery in medially tight knees.

## Data Availability

Not applicable.
